# Melatonin Mitigates Cisplatin-Induced Ovarian Dysfunction via Altering Steroidogenesis, Inflammation, Apoptosis, Oxidative Stress, and PTEN/PI3K/Akt/mTOR/AMPK Signaling Pathway in Female Rats

**DOI:** 10.3390/pharmaceutics14122769

**Published:** 2022-12-10

**Authors:** Amal Al-Shahat, Mohey A. E. Hulail, Nada M. M. Soliman, Tarek Khamis, Liana Mihaela Fericean, Ahmed Hamed Arisha, Rania S. Moawad

**Affiliations:** 1 Human Anatomy & Embryology Department, Faculty of Medicine, Zagazig University, Zagazig 44519, Egypt; 2 Department of Pharmacology, Faculty of Veterinary Medicine, Zagazig University, Zagazig 44519, Egypt; 3 Laboratory of Biotechnology, Faculty of Veterinary Medicine, Zagazig University, Zagazig 44519, Egypt; 4 Biology Department, Faculty of Agriculture, University of Life Sciences “King Michael I of Romania” from Timisoara, Aradului St. 119, 300645 Timisoara, Romania; 5 Department of Animal Physiology and Biochemistry, Faculty of Veterinary Medicine, Badr University in Cairo (BUC), Badr City 11829, Egypt; 6 Department of Physiology, Faculty of Veterinary Medicine, Zagazig University, Zagazig 44511, Egypt

**Keywords:** cisplatin, melatonin, ovary, steroidogenesis, oxidative stress, ovarian impairment

## Abstract

Ovarian damage and fertility impairment are major side effects of chemotherapy in pre-menopausal cancer patients. Cisplatin is a widely used chemotherapeutic drug. The present study was designed to assess the ameliorative effects of melatonin as an adjuvant for fertility preservation. Thirty-two adult female Wistar rats were divided randomly into four equal groups: Control, Melatonin, Cisplatin (CP) treated, and CP + Melatonin treated. The cisplatin-treated group showed decreased body and ovarian weights, decreased serum E2 and AMH, increased serum LH and FSH, reduced ovarian levels of SOD, CAT, GSH, and TAC, and increased ovarian MDA. The histopathological examination of the cisplatin-treated group showed deleterious changes within ovarian tissue in the form of damaged follicles and corpus luteum, hemorrhage, and inflammatory infiltrates with faint PAS reaction in zona pellucida, increased ovarian collagen deposition, and marked expression of caspase-3 immune reaction in granulosa and theca cells, stroma, and oocytes. Alongside, there was a significant downregulation in the mRNA expression of steroidogenic enzymes, IL10, AMPK, PI3K, AKT, mTOR, and PTEN, while TGF-β1, IL1β, IL6, TNF-α, NF-Kβ, P53, p38-MAPK, JNK, and FOXO3 mRNA expressions were upregulated in cisplatin-treated rats’ ovarian tissue. Coadministration of cisplatin-treated rats with melatonin reversed these changes significantly. In conclusion, melatonin’s antioxidant, anti-inflammatory, and anti-apoptotic activities could modulate ovarian disturbances induced by cisplatin and preserve fertility.

## 1. Introduction

The international agency for research on cancer in 2018 stated that more than 276,000 people in Europe under the age of 44 suffered from cancer. At these young age groups, the incidence rates are 65% for females and 35% for males. While current cancer treatment approaches are frequently able to provide disease remission to prolong patients’ life expectancy, they may also lead to premature ovarian failure and infertility owing to the extreme sensitivity of ovarian follicles to the injurious effects of chemotherapeutic drugs. Such fertility impairment could be psychologically traumatic for women, as most of them may desire to have biological children [1]. Therefore, fertility preservation is becoming a clinical and moral duty in oncological practice [2]. Using gonadal-protective agents may be convenient as selected to improve the efficacy of fertility preservation and reproductive health in chemotherapy patients [3].

Cisplatin (cis-diaminodichloroplatin II, CP) is one of the most powerful antineoplastic drugs used against a wide spectrum of malignancies. It acts on cancer cells by forming adducts and damage of DNA resulting in apoptosis and cell cycle arrest [4]. In addition, CP can internalize in organelles, such as the endoplasmic reticulum, mitochondria, lysosomes, and nucleus, impairing their homeostasis [5]. Yet, anticancer medications could have adverse cytotoxic effects on healthy cells and tissues including the reproductive system. Chemotherapy can cause changes in fertility, organ structure, sexual hormones, and function and even produce fertility loss, thus decreasing the quality of life [6,7].

Melatonin (N-acetyl-5-methoxytryptamine) is primarily synthesized and secreted by the pineal gland in animals and humans [8]. Peripheral organs, including the retina, kidney, testes, ovary, and vasculature, as well as immune cells also synthesize melatonin. Melatonin adjusts a variety of physiological functions, such as circadian rhythm and clock genes, mood, sexual behavior, the immune system, and body temperature and blood pressure regulation. In addition, melatonin is involved in regulating the reproductive cycle (i.e., estrous cycle in animals), follicular and blastocyst development, and embryo implantation [9]. Melatonin could regulate several molecular pathways, underlying inflammation, oxidative stress, proliferation, apoptosis, metastasis, and autophagy in different pathophysiological situations [10,11].

In the current work, the cisplatin exposure negative effects on the ovary of adult female rats were evaluated and the potential role of melatonin adjuvants to preserve fertility was examined.

## 2. Materials and Methods

### 2.1. Chemicals and Reagents

Cisplatin was obtained as a vial for injection (50 mg/50 mL, Mylan, Italy) from a local pharmacy (Egypt) as it is commercially available. Melatonin powder (50 mg melatonin/mL ethanol) was purchased (purity ≥ 98%, CAS 73-31-4, Sigma Aldrich, St. Louis, MO, USA).

### 2.2. Animals

Thirty-two adult female Wistar rats (180–200 g, 3 months old) with a normal estrus cycle (4–5) days determined by daily vaginal smears as described previously [12] were used in this study. Rats were obtained from the animal house facility of Zagazig University. The rats were group housed at the rate of three per cage, maintained in a 12 h light/dark cycle at a constant temperature (24 ± 1 °C), and fed a standard rodent pellet diet. Tap water was provided ad libitum. All rats received humane care in compliance with the guidelines of the Ethical Committee of Zagazig University and in accordance with the National Institutes of Health (NIH) Guidelines for the Care and Use of Laboratory Animals, and the experimental methods were approved by the Institutional Animal Care and Use Committee of Zagazig University (ZU-IACUC/3/F/171/2019), Egypt.

### 2.3. Experimental Design and Sample Collection

Rats were divided randomly into four equal groups (8 each) as follows: Group I (control group) received intraperitoneal normal saline and ethanol mixture (4.4 mL/kg and 0.6 mL/kg respectively) once daily for seven days. Group II (Melatonin group) received melatonin (30 mg/kg body weight; i.p) once a day for seven days [13]. Melatonin solution (5 mL/kg) was prepared as follows; firstly, 30 mg melatonin was dissolved in 0.6 mL/kg absolute ethanol (50 mg melatonin/mL ethanol), then diluted with normal saline (4.4 mL/kg). Group III (CP-treated group) received a single dose of CP (7 mg/kg of body weight; i.p) (7 mL/kg) on the first day [14]. Group IV (CP+ melatonin group) received melatonin (30 mg/kg body weight; i.p) (5 mL/kg) once a day for seven days, along with the single dose of CP (7 mg/kg body weight; i.p) (7 mL/kg) on the first day. Melatonin was administered at 10:00 a.m. Additionally, to evade the potential influence of hormonal changes at different stages of the estrous cycle, the estrous cycle of the female rat was monitored, and all rats were injected at the estrous stage.

All animals were weighed and anesthetized by intraperitoneal injection of 75 mg/kg sodium thiopental on the 8th day. Then, blood samples were immediately collected from the rat tail for hormonal assay. Blood samples were centrifuged at 2500 rpm to separate the serum. Then, serum was stored at −80 °C until the measurement date. Laparotomy was done, and then the ovaries were excised carefully and weighed. The right ovary was processed for light-microscopic examination and the left ovary was flash-frozen and kept at −80 °C until used for biochemical and molecular analysis.

### 2.4. Biochemical Analysis

Serum concentrations of luteinizing hormone (LH), follicular stimulating hormone (FSH), estradiol (E2), and anti-Mullerian hormone (AMH) were estimated by rat enzyme- linked immunosorbent assay (ELISA) kits (Cusabio Biotech Co., Ltd., Wuhan, China) following the manufacturer’s instructions.

The ovarian tissue was homogenized in a Teflon-glass homogenizer using a buffer of 1.15% potassium chloride (KCl) to get a 1/10 (*w*/*v*) homogenate. Homogenates were centrifuged at 4000 rpm (+4 °C) for 15 min for the determination of catalase (CAT), malondehyde (MDA), reduced glutathione (GSH), superoxide dismutase (SOD), and total antioxidant capacity (TAC) levels by bio diagnostic kits obtained from Diagnostic and Research Reagents (Giza, Egypt) following the manufacturer’s instructions.

### 2.5. Light Microscopic Analysis

According to standard procedures, the right ovaries were immersed in 10% neutral buffered formalin and embedded in paraffin. Serial sections of 5 μm thick at every 5th section were mounted on glass slides, deparaffinized with xylene, and then stained with the following stain; hematoxylin and eosin (H&E) to investigate the distribution of ovarian follicles in the ovaries, periodic acid Schiff (PAS) reagent to detect carbohydrates stores and the integrity of zona pellucida and oocyte, and Masson’s trichrome (MT) stain to detect the amount of collagen deposition within ovarian tissue [15,16].

### 2.6. Immunohistochemical Analysis

Ovarian tissue expression of active caspase-3 was done via immunostaining using the avidin–biotin complex technique [17]. Paraffin sections were deparaffinized in xylene and rehydrated by a series of graded alcohols. Boiling the sections in 0.1 M sodium citrate to perform antigen retrieval. Sections were treated with 3% hydrogen peroxidase to inhibit endogenous peroxidase activity. The sections were then incubated with 10% normal goat serum for 1 h at room temperature to block the non-specific binding of antibodies. Then the sections were incubated with primary antibodies targeting active caspase-3 (Novus, rabbit polyclonal IgG, cat. no1-CA222-02) at 4 °C overnight. The sections were washed 3 times with PBS and the biotinylated secondary antibody was applied for 20 min. Then, sections were washed with PBS before incubating (20 min) with the streptavidin peroxidase kit (Vector Laboratories). The sections were finally developed using 3,3-diaminobenzidine tetrahydrochloride kit (DAB, Vector Laboratories) and counterstained with Mayer’s hematoxylin, and examined by a light microscope. A positive reaction was indicated by brown cytoplasmic and nuclear staining of the cells and oocytes. All stained slides were examined, and images of the histological sections were obtained using a light microscope fitted with a digital camera (The Leica DM500, Leica ICC50 W Camera Module, Anatomy Department, Faculty of Medicine, Zagazig University).

### 2.7. Morphometric Analysis

For the morphometrical study, image J 1.49v/Java 1.6.0_244” software analyzer computer system (National Institutes of Health, Bethesda, MD, USA) was used. The number of ovarian follicles was estimated by using the measuring field menu in H&E slides of the ovary in random microscopic areas under 100 high power fields. A mean of 15 readings was estimated from 5 serial sections from slides of each animal in each group (the total was 120 measurements/group). H&E staining was applied on every 20th section of the ovary so that each section was separated by a distance of approximately 50–60 μm from the next 20th section to avoid counting the same follicle twice [18]. According to [19], only oocyte-containing ovarian follicles were considered and follicles were classified as primordial, primary, secondary, and Graafian follicles.

In addition, the PAS optical density and the caspase-3 positive immune reaction area percent were measured in PAS-stained and caspase-3 immune-stained sections, respectively, within ten high power field readings that were obtained from 5 serial sections from slides of each specimen for each rat at a magnification ×400. (The total was 80 measurements/group). The image analyzer was first calibrated automatically to convert the measurement units (pixels) produced by the image analyzer program into actual micrometer units and data was recorded and presented as mean ± standard deviation and processed for statistical analysis.

### 2.8. Real-Time Quantitative RT-PCR (qRT-PCR) Analysis

According to [20] briefly, 30 mg of ovarian tissue have been used from which total RNA was extracted using Trizol (Invitrogen; Thermo Fisher Scientific, Waltham, MA, USA), and for evaluating the RNA quality, the A260/A280 ratio was analyzed using the Nano DropVR ND-1000 Spectrophotometer (Nano Drop Technologies, Wilmington, DE, USA) for 1.5 mL of the RNA. For cDNA synthesis, a High-Capacity cDNA Reverse Transcription Kit cDNA Kit; (Applied Biosystems™, Waltham, MA, USA) was used, followed by the preparation of the primers according to their manufacturer instructions Sangon Biotech (Beijing, China) as provided in Table 1.

Real-time RT-PCR was performed in Mx3005P real-time PCR system (Agilent Stratagene Technologies, Inc., Santa Clara, CA, USA) using TOPrealTM qPCR 2X PreMIX (SYBR Green with low ROX) (Cat. # P725 or P750) (Enzynomics, Daejeon, Republic of Korea) following the manufacturer’s instructions. The PCR cycling conditions included initial denaturation at 95 °C for 12 min followed by 40 cycles of denaturation at 95 °C for 20 s, annealing at 60 °C for 30 s, and extension at 72 °C for 30 s. A melting curve analysis was performed following PCR amplification. The expression level of the target genes was normalized using the mRNA expression of a known housekeeping gene *Gapdh*. Results are expressed as fold-changes compared to the control group following the 2^−ΔΔCt^ method [21].

### 2.9. Statistical Analysis

Continuous variables were represented by the mean ± standard error mean (SEM). Data were analyzed by one-way analysis of variance (ANOVA) followed by Tukey’s honest significant difference test in homogenous data for multiple group comparison. *p* value less than 0.05 (*p*  <  0.05) was considered to be statistically significant using the statistical software package SPSS for Windows (Version 20; SPSS Inc., Chicago, IL, USA) [22].

## 3. Results

### 3.1. Body Weight, the Weight of Paired Ovary, and Serum Hormones

**Figure 1:**
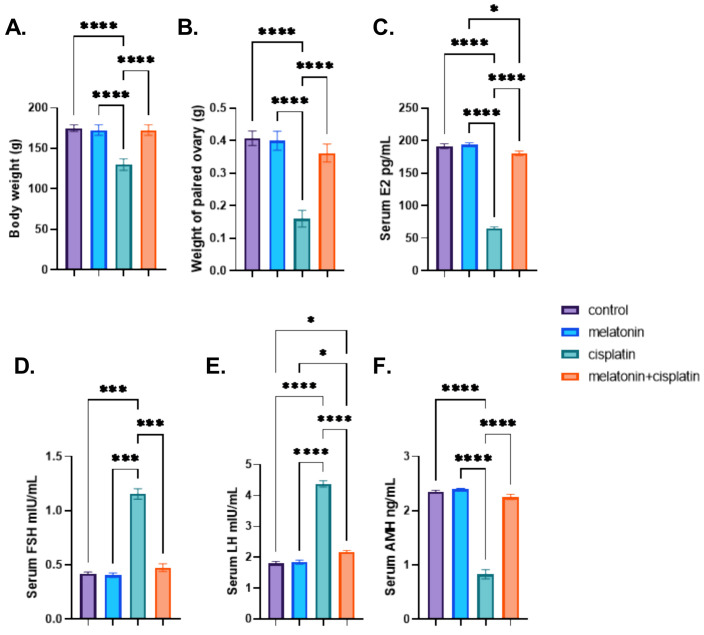
Effect of melatonin administration in cisplatin-induced ovarian impairment in female rats on body weight, the weight of paired ovary, and serum hormones (**A**–**E**). (**A**) body weight (g), (**B**) weight of paired ovary (g), (**C**) serum E2 level (pg/mL), (**D**) serum FSH (mIU/mL) level, (**E**) serum LH (mIU/mL) level, and (**F**) serum AMH (ng/mL) level. Data are expressed as means ± SEM. *n* = 8. *, ***, **** indicate significant difference (*p* < 0.05, *p* < 0.001, and *p* < 0.0001).

The present study demonstrated a non-significant difference between different groups with regard to initial body weight. Administration of cisplatin caused a highly significant decrease in body weight and weight of paired ovary compared to the control group. Co-administration of melatonin with cisplatin resulted in a highly significant increase in body weight and weight of paired ovary compared to the cisplatin-treated group. There was no statistically significant difference between the control group and the melatonin group in Figure 1A,B.

The cisplatin-treated group showed a highly significant increase in FSH and LH levels and a highly significant reduction in both serum E2 and AMH levels compared to the control group. Cisplatin and melatonin group there was a highly significant decrease in serum levels of FSH and LH and a highly significant increase in serum E2 and AMH levels compared to the cisplatin-treated group. There was no statistically significant difference between the control group and the melatonin group in Figure 1C–F.

### 3.2. Light Microscopic Analysis of the Ovary

#### 3.2.1. Ovarian Hematoxylin and Eosin (H&E) 

**Figure 2:**
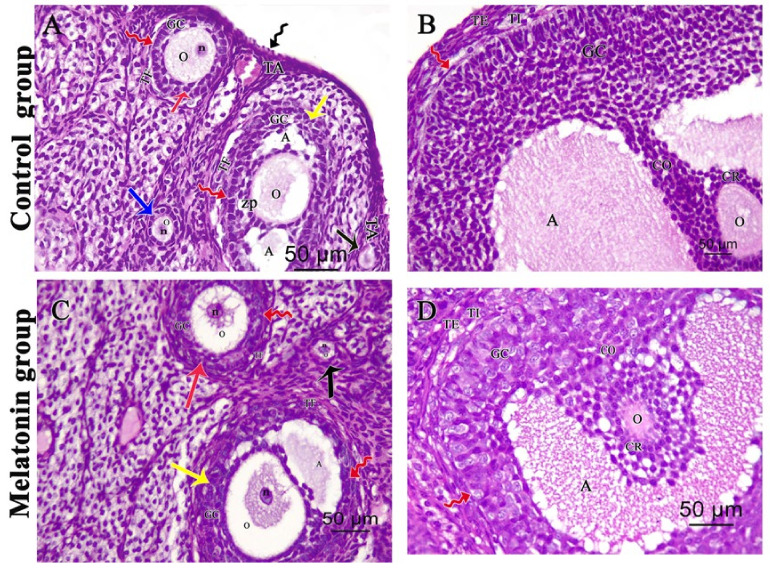
Photomicrographs of (H&E ×400) stained ovarian sections of (**A**,**C**) showing surface epithelium (black zigzag arrow), tunica albuginea (TA), primordial follicle (black arrow), unilaminar primary follicle (blue arrow) with oocyte (O) having nucleus (n) and multilaminar primary follicle (red arrow) formed of granulosa cells (GC) and oocyte (O) having clear nucleus (n). Secondary follicle (yellow arrow) with antral cavity (A), an oocyte (O), and zona pellucida (ZP). Stroma around both multilaminar and secondary follicles form theca folliculi (TF) separated from granulose cells by a basement membrane (red zigzag arrow). (**B**,**D**) showing: Graafian follicle with an oocyte (O), corona radiata (CR), and cumulus oophorous (CO) that connects the oocyte with the rest of the granulosa cells (GC). Follicular antrum (A), basement membrane (red zigzag arrow), and theca folliculi with two layers theca interna (TI) and theca externa (TE) are also seen.

Examination of H&E-stained ovarian sections of the control and melatonin group revealed a normal ovarian histological structure with outer simple surface epithelium with tunica albuginea beneath it. An ovarian cortex is characterized by multiple follicles in various stages of development embedded in connective tissue stroma, as well as normal oocyte and corpus luteum in Figure 2A,C. Graafian follicle had an oocyte, corona radiata, and cumulus oophorous that connects the oocyte with the rest of the granulosa cells in Figure 2B,D.

**Figure 3:**
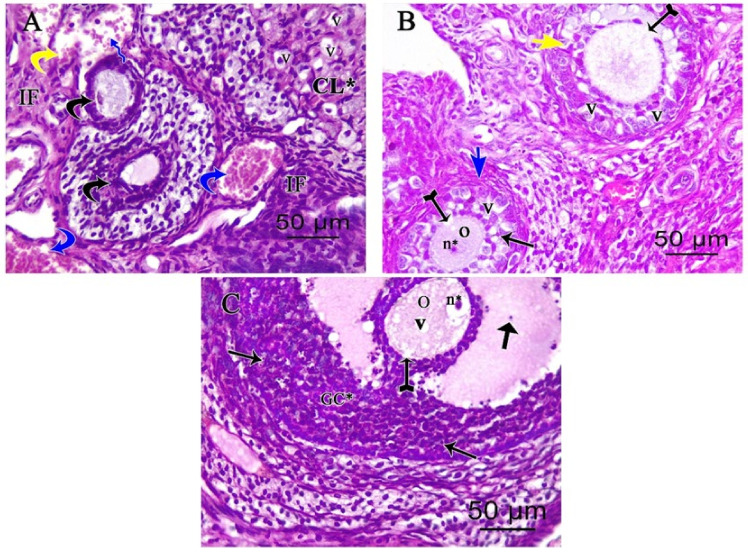
Photomicrographs of (H&E ×400) stained ovarian sections of cisplatin-treated group showing (**A**) degenerating follicles (black curved arrows), dilated congested (blue curved arrows), and ruptured (blue zigzag arrow) blood vessels. Hemorrhage (yellow curved arrows), inflammatory infiltrate (IF), and corpus luteum (CL*) with cell vacuolations (v) are also seen. (**B**) Degenerated multilaminar primary follicle (short blue arrow) with oocyte having pyknotic nucleus (n*), ruptured faint zona pellucida (tailed arrow), and granulosa cells with vacuolation (v) and pyknotic nuclei (thin arrow). A secondary follicle (short yellow arrow) with ruptured faint zona pellucida (tailed arrow) and vacuolated (v) disorganized granulosa cells was observed. (**C**) Degenerating Graafian follicle with an oocyte (O) showing cytoplasmic vacuolations (V) and micronuclei (n*), faint ruptured zona pellucida (tailed arrow), disorganized granulosa cells (GC*) with pyknotic nuclei (thin long arrows). Floating granulosa cells within an antral cavity (thick arrows).

Examination of the cisplatin-treated group showed a marked loss of normal ovarian architecture. Severe follicular degeneration was observed in different stages of development with inflammatory cellular infiltrates, vacuolations in the corpus luteum, dilated congested blood vessels, and hemorrhage in Figure 3A. Degenerated multilaminar follicles had oocytes with pyknotic nuclei and ruptured faint zona pellucida with vacuolated granulosa cells in Figure 2B. Degenerated Graafian follicle had vacuolated oocyte and micronucleus with faint ruptured zona pellucida and disorganized granulosa cells with pyknotic nuclei. Some floating granulosa cells within the antral cavity were observed in Figure 2C.

**Figure 4:**
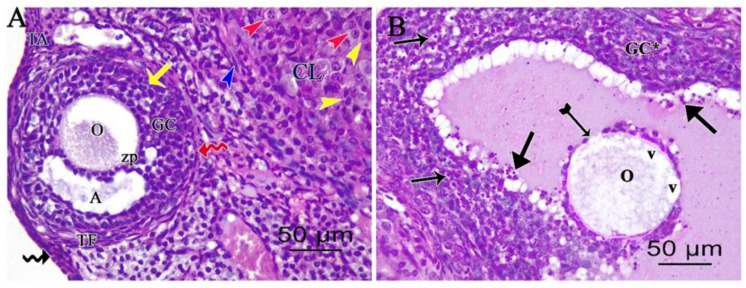
Photomicrographs of (H&E ×400) stained ovarian sections of cisplatin and melatonin-treated group showing: (**A**) Intact surface epithelium (zigzag black arrow) with tunica albuginea (TA) beneath. Corpus luteum (CL) with theca lutein (yellow arrowhead), granulosa lutein cells (red arrowhead), and theca externa cells (blue arrowhead) are seen. The secondary follicle (yellow arrow) is formed of multiple layers of organized granulosa cells (GC) with an antral cavity (A), an oocyte (O), zona pellucida (ZP), basement membrane (red zigzag arrow), and theca folliculi (TF). (**B**) Degenerated Graafian follicle has an oocyte (O) with cytoplasmic vacuolations (V), faint ruptured zona pellucida (tailed arrow), and disorganized granulosa cells (GC*) with pyknotic nuclei (thin long arrows). Some floating granulosa cells (thick arrows) within the antral cavity are still seen.

The cisplatin and melatonin-treated group revealed the presence of a nearly normal ovarian histological structure. The secondary follicle had multiple layers of organized granulosa cells with an antral cavity and oocyte. Corpus luteum with theca lutein, granulosa lutein cells, and theca externa cells are seen in Figure 4A. Degenerated Graafian follicle was still seen showed an oocyte with cytoplasmic vacuolations, faint ruptured zona pellucida and disorganized granulosa cells with pyknotic nuclei. Some floating granulosa cells within the antral cavity were still seen in Figure 4B.

#### 3.2.2. Ovarian Periodic Acid-Schiff Reagent (PAS)

**Figure 5:**
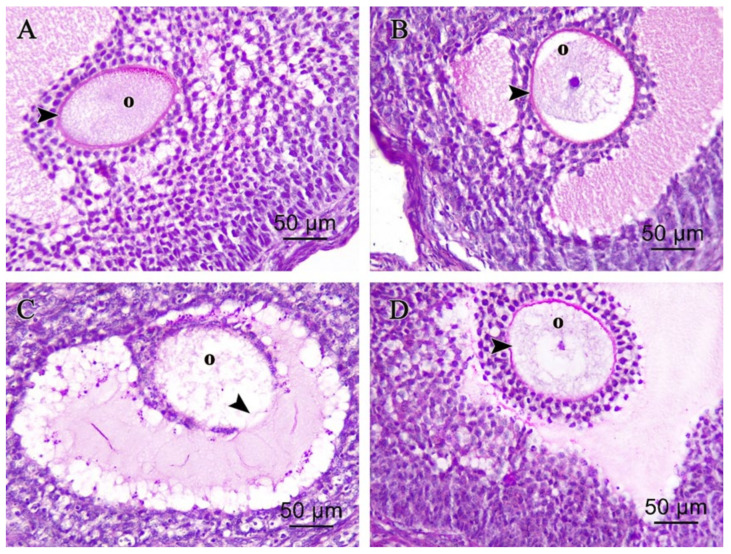
Photomicrographs of (PAS ×400) stained ovarian sections. (**A**) Control group, and (**B**) melatonin group showing: Graafian follicle displayed positive PAS reactions of intact continuous zona pellucida (arrowhead) surrounding intact oocyte (O). (**C**) Cisplatin-treated group showed degenerating Graafian follicle with faint PAS reactions in damaged interrupted zona pellucida (black arrowhead) and oocyte (O). (**D**) Cisplatin + melatonin-treated group showing: Graafian follicle displayed positive PAS reactions of intact continuous zona pellucida (arrowhead) surrounding intact oocyte (O).

Control and melatonin PAS-stained ovarian sections showed Graafian follicle with positive PAS reactions in intact continuous zona pellucida surrounding the oocyte in Figure 5A,B respectively. In the cisplatin-treated group, PAS-stained ovarian sections showed damaged zona pellucida and oocyte with faint PAS reactions in Figure 5C. In the melatonin and cisplatin-treated group, PAS-stained ovarian sections showed Graafian follicle with positive PAS reactions in intact continuous zona pellucida surrounding the oocyte in Figure 5D.

#### 3.2.3. Ovarian Masson’s Trichrome (MT) Stain

**Figure 6:**
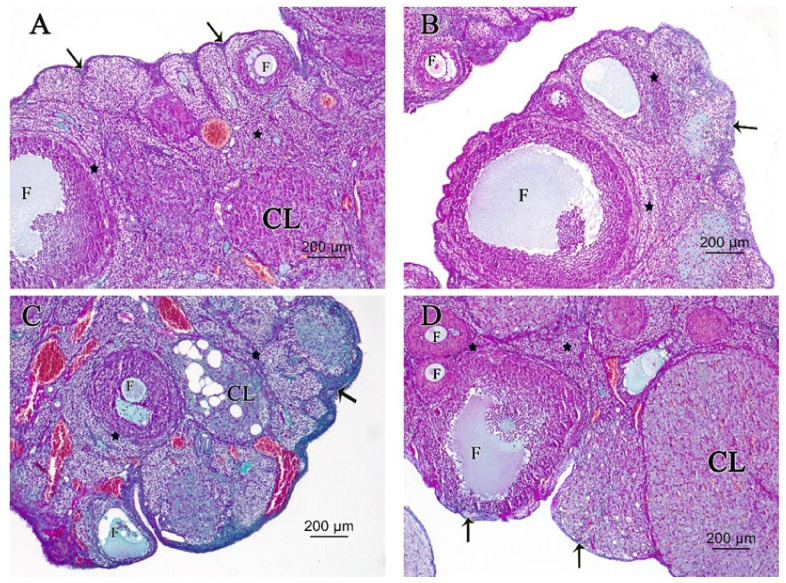
Photomicrographs of (MT ×100) stained ovarian sections. (**A**) Control group and (**B**) melatonin group showing normal deposition of delicate collagen fibers in tunica albuginea (arrow) and stroma (star) around and in between follicles (F). (**C**) Cisplatin-treated group showed increased deposition of collagenous fibers in tunica albuginea (arrow) and the stroma (star) between follicles (F) and Corpus Luteum (CL). (**D**) Cisplatin + melatonin-treated group showed normal deposition of delicate collagen fibers in tunica albuginea (arrow) and the stroma (star) around and in between follicles (F) and Corpus Luteum (CL).

Control and melatonin MT-stained ovarian sections in Figure 6A,B, respectively, revealed normal density and distribution of delicate collagen fibers in tunica albuginea and stroma around follicles, while increased collagenous deposition in tunica albuginea beneath the surface epithelium and the stroma surrounding follicles in cisplatin-treated rats can be seen in Figure 6C. Melatonin co-treatment ameliorated increased deposition of collagenous fibers in tunica albuginea beneath the surface epithelium and the stroma surrounding follicles in cisplatin-treated rats in Figure 6D.

### 3.3. Caspase-3 Immunohistochemical Staining

**Figure 7:**
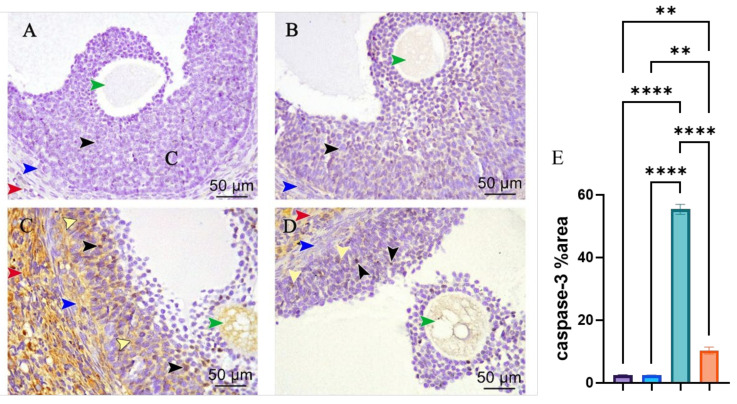
Photomicrographs of Caspase 3 immune reaction (IHC staining × 400) in ovarian sections (**A**–**E**). (**A**) Control group and (**B**) melatonin group showing Graafian follicle with negative immune reactions in the granulosa (black arrowhead), theca cells (blue arrowhead), stroma (red arrowhead), and the oocyte (green arrowhead). (**C**) Cisplatin-treated group showing degenerating Graafian follicle with a positive immune reaction in the majority of granulosa (nuclear, black arrowheads and cytoplasmic, yellow arrowheads) and in theca cells (blue arrowheads) besides the positive reactions of the oocyte (green arrowhead). Moreover, extensive positive immune reactions in the stroma (red arrowhead). (**D**) Cisplatin + melatonin group showing Graafian follicle with a few granulosa cells with positive immune reactions (nuclear reaction, black arrowhead and cytoplasmic reaction, yellow arrowhead). Few theca cells with positive immune reactions (blue arrowhead) besides the mild positive reactions of the oocyte (green arrowhead) and stroma (red arrowhead) are observed. (**E**) Immunostaining intensity of ovarian caspase-3 (% area). **, and **** indicate significant difference (*p* < 0.01, and *p* < 0.0001).

Caspase-3-stained ovarian sections of control and melatonin in Figure 7A,B, respectively, group displayed negative immunoreaction for caspase-3 in the granulosa, theca cells, oocyte of Graafian follicles, and stroma, while the cisplatin-treated group showed Graafian follicles with positive immune reactions in the majority of the granulosa and theca cells besides the positive reaction of the oocyte and extensive positive reaction in the stroma in Figure 7C. The cisplatin and melatonin-treated group displayed Graafian follicle with a few positive immune reactions in the granulosa and theca cells besides the mild positive reactions of the oocyte and stroma in Figure 7D. There was a highly statistically significant increase in the area percent of immune-expression of caspase-3 in the cisplatin-treated group compared to the control group, whereas there was a highly statistically significant decrease in the cisplatin and melatonin groups. There was no statistically significant difference between the control group and the melatonin group in Figure 7E.

### 3.4. Morphometric Studies; Ovarian Follicles Counting

**Figure 8:**
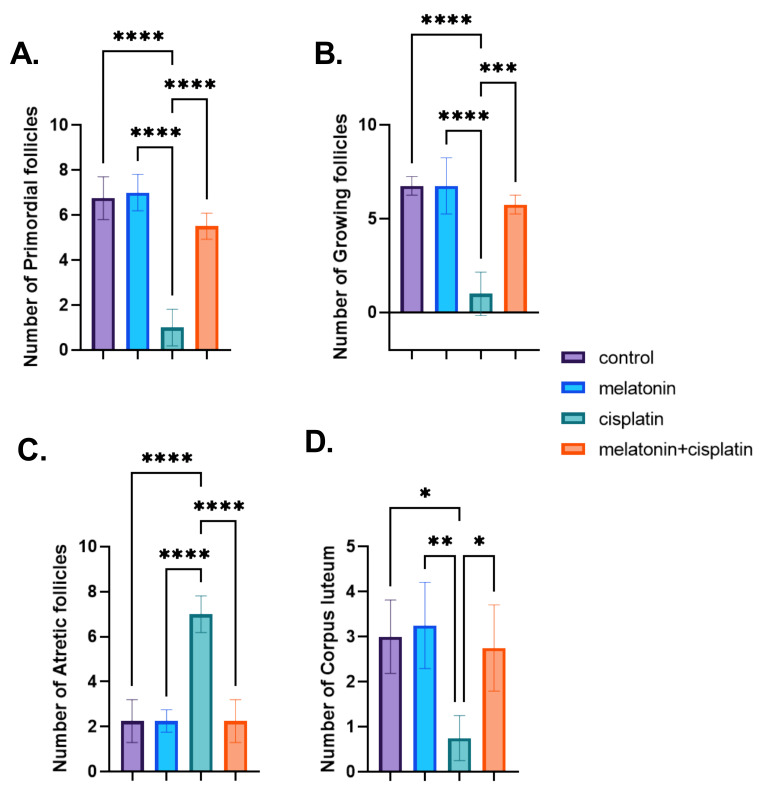
Effect of melatonin administration in cisplatin-induced ovarian impairment in female rats on ovarian follicles counting (**A**–**D**). (**A**) The number of primordial follicles, (**B**) number of growing follicles, (**C**) number of atretic follicles, and (**D**) number of corpus luteum. Data are expressed as means ± SEM. *n* = 8. *, **, ***, **** indicate significant difference (*p* < 0.05, *p* < 0.01, *p* < 0.001, and *p* < 0.0001).

The cisplatin-treated group showed a highly statistically significant decrease in the numbers of primordial and growing follicles and size/number of corpus luteum and a highly statistically significant increase in the numbers of atretic follicles compared to the control group in Figure 8A–D, whereas there was a highly statistically significant increase in the numbers of primordial and growing follicles and size/number of corpus luteum and a highly significant decrease in the numbers of atretic-follicles in the cisplatin and melatonin-treated group. There was no statistically significant difference between the control group and the melatonin group in Figure 8A–D.

### 3.5. Ovarian Oxidative Stress Biomarkers

**Figure 9:**
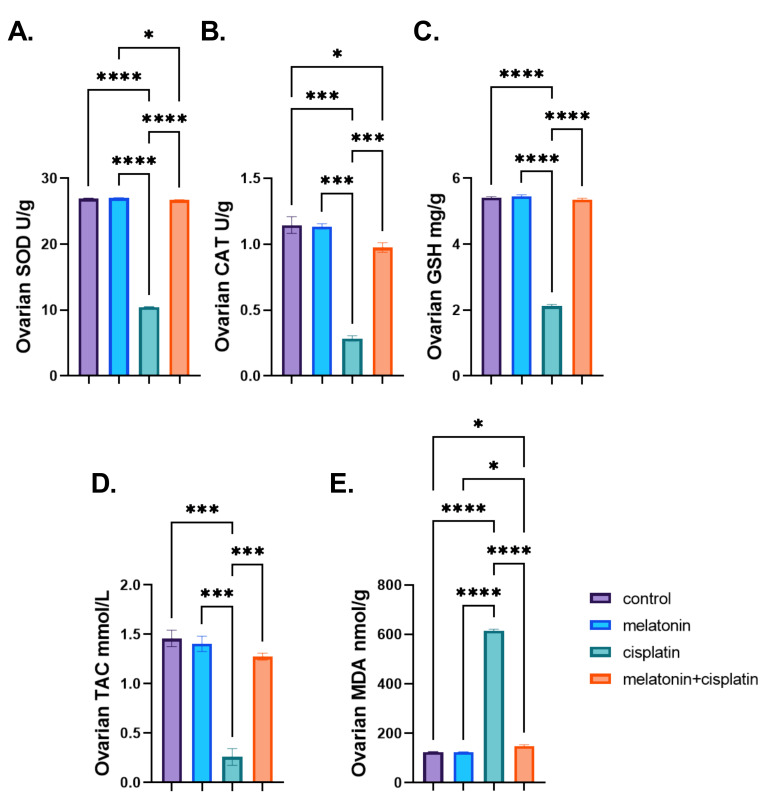
Effect of melatonin administration in cisplatin-induced ovarian impairment in female rats on ovarian oxidative stress biomarkers (**A**–**E**). (**A**) Ovarian SOD (U/g), (**B**) ovarian CAT (U/g), (**C**) ovarian GSH (mg/g), (**D**) ovarian TAC (mmol/L), and (**E**) ovarian MDA (nmol/g). Data are expressed as means ± SEM. *n* = 8. *, ***, **** indicate significant difference (*p* < 0.05, *p* < 0.001, and *p* < 0.0001).

There was a highly significant decrease in ovarian levels of antioxidant enzymes (SOD and CAT), non-enzymatic (GSH), and TAC in the cisplatin-treated group while there was a highly significant increase in the ovarian levels of MDA in the same group as compared to the control group, andle there was a highly significant increase in ovarian levels of antioxidant enzymes (SOD and CAT), non-enzymatic (GSH), and TAC in cisplatin and melatonin-treated group and a highly significant decrease in MDA ovarian level in the same group as compared to the cisplatin-treated group. There was no statistically significant difference between the control group and the melatonin group in Figure 9A–E.

### 3.6. mRNA Expression of Ovarian Steroidogenic Pathway

**Figure 10:**
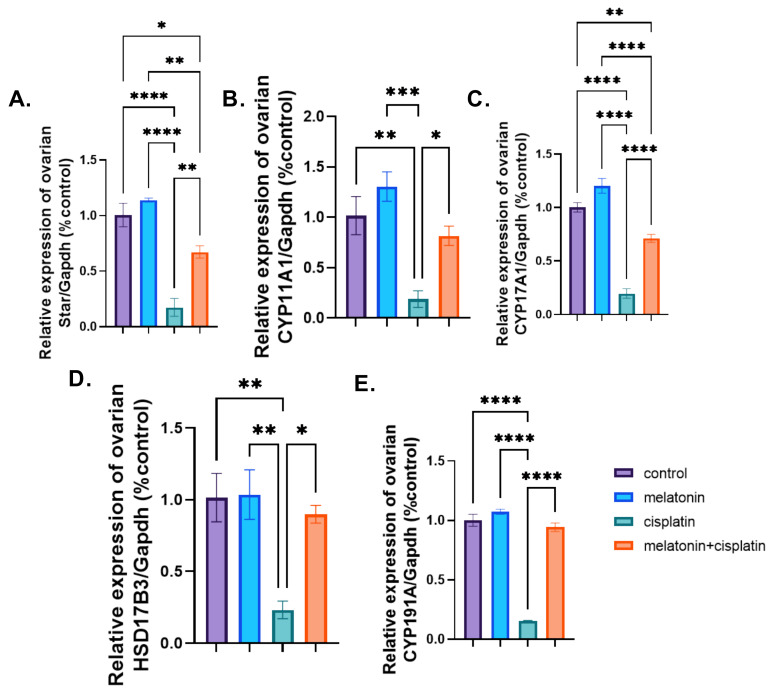
Effect of melatonin administration in cisplatin-induced ovarian impairment in female rats on mRNA expression of ovarian Cyp19A1, Cyp17A1, Cyp11a1, HSD17B3, and STAR enzymes (**A**–**E**). (**A**). Ovarian Star/*Gapdh* (% control), (**B**) ovarian Cyp11a1/*Gapdh* (% control), (**C**) ovarian Cyp17A1/*Gapdh* (% control), (**D**) ovarian HSD17B3/*Gapdh* (% control), and (**E**) ovarian Cyp19A1/*Gapdh* (% control). Data are expressed as means ± SEM. *n* = 8. *, **, ***, **** indicate significant difference (*p* < 0.05, *p* < 0.01, *p* < 0.001, and *p* < 0.0001).

mRNA expression of Cyp19A1, Cyp17A1, Cyp11a1, HSD17B3, and STAR:

There was a highly significant downregulation of mRNA expression of steroidogenesis (Cyp19A1, Cyp17A1, Cyp11a1, HSD17B3, and STAR) in the cisplatin-treated group as compared to the control group. Co-administration of melatonin with cisplatin resulted in a highly significant upregulation of genes expression of steroidogenesis (Cyp19A1, Cyp17A1, Cyp11A1, HSD17B3, and STAR) compared to the cisplatin-treated group. There was no statistically significant difference between the control group and the melatonin group in Figure 10A–E.

### 3.7. mRNA Expression of Proinflammatory and Anti-Inflammatory Markers

**Figure 11:**
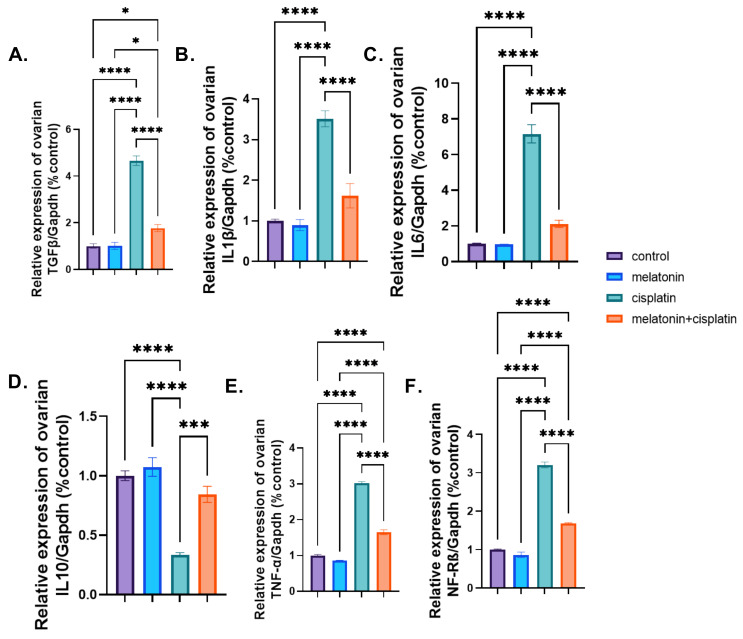
Effect of melatonin administration in cisplatin-induced ovarian impairment in female rats on mRNA expression of ovarian proinflammatory and anti-inflammatory marker genes (**A**–**F**). (**A**) Ovarian TGF-β1/*Gapdh* (% control), (**B**) ovarian IL1β/*Gapdh* (% control), (**C**) ovarian IL6/*Gapdh* (% control), (**D**) ovarian IL10/*Gapdh* (% control), (**E**) ovarian TNF-α /*Gapdh* (% control), and (**F**) ovarian NF-Kβ /*Gapdh* (% control). Data are expressed as means ± SEM. *n* = 8. *, ***, **** indicate significant difference (*p* < 0.05, *p* < 0.001, and *p* < 0.0001).

The mRNA expression of pro-inflammatory markers (IL1β, IL6, TNF-α, NF-Kβ, and TGF-β1) revealed a highly significant upregulation, while the anti-inflammatory marker IL10 showed a highly significant downregulation in the cisplatin-treated group, in the comparison with the control group in Figure 11A–F. Meanwhile, co-treatment of melatonin and cisplatin resulted in a highly significant downregulation of genes expression of pro-inflammatory markers (IL1β, IL6, TNF-α, and NF-Kβ and upregulation of gene expression of an anti-inflammatory marker, IL10, when compared to the cisplatin-treated group. There was no statistically significant difference between the control group and the melatonin group in Figure 11A–F.

### 3.8. mRNA Expression of PI3K-Akt/mTOR/AMPK Pathway

**Figure 12:**
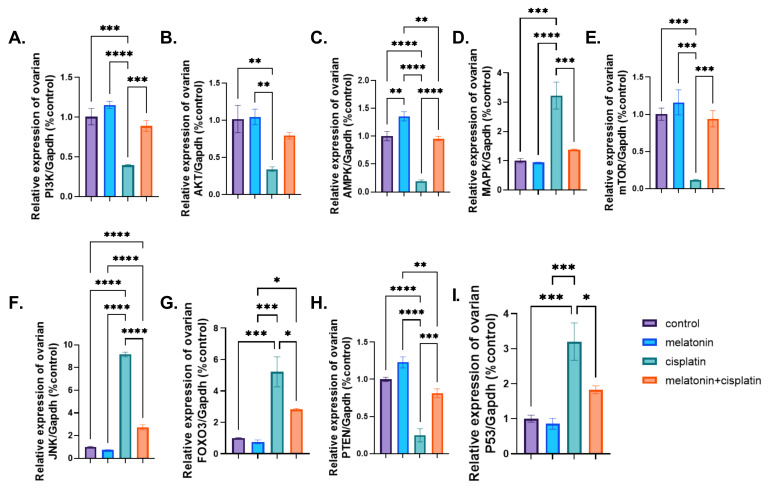
Effect of melatonin administration in cisplatin-induced ovarian impairment in female rats on ovarian PI3K-Akt/mTOR/AMPK signaling pathway (**A**–**I**). (**A**) Ovarian PI3K/*Gapdh* (% control), (**B**) ovarian Akt/*Gapdh* (% control), (**C**) ovarian AMPK/*Gapdh* (% control), (**D**) ovarian MAPK/*Gapdh* (% control), (**E**) ovarian mTOR/*Gapdh* (% control), (**F**) ovarian JNK/*Gapdh* (% control), (**G**) ovarian FOXO3/*Gapdh* (% control), (**H**) ovarian PTEN/*Gapdh* (% control), and (**I**) ovarian P53/*Gapdh* (% control). Data are expressed as means ± SEM. *n* = 8. *, **, ***, **** indicate significant difference (*p* < 0.05, *p* < 0.01, *p* < 0.001, and *p* < 0.0001).

Treatment of rats with cisplatin caused a highly significant downregulation of PI3k, AKT, mTOR, and PTEN mRNA expression, and a highly significant upregulation of FOXO3 gene expression compared to the control group. Co-administration of melatonin with cisplatin resulted in a highly significant upregulation of PI3k, AKT, mTOR, and PTEN genes expression and a highly significant downregulation of FOXO3 compared to the cisplatin-treated group in Figure 12A,B,E,G,H. There was no statistically significant difference between the control group and the melatonin group in Figure 12A,B,E,G,H.

Treatment of rats with cisplatin caused a highly significant upregulation of AMPK mRNA expression compared to the control group. Co-administration of melatonin with cisplatin resulted in a highly significant downregulation of AMPK mRNA expression compared to the cisplatin-treated group in Figure 12C. There was no statistically significant difference between the control group and the melatonin group in Figure 12C.

Treatment of rats with cisplatin caused a highly significant upregulation of p38- MAPK and JNK mRNA expression compared to the control group. Co-administration of melatonin with cisplatin resulted in a highly significant downregulation of p38-MAPK and JNK mRNA expression compared to the cisplatin-treated group in Figure 12D,F. There was no statistically significant difference between the control group and the melatonin group in Figure 12D,F.

Treatment of rats with cisplatin caused a highly significant upregulation of tumor suppressor P53 gene expression compared to the control group. Co-administration of melatonin with cisplatin resulted in a highly significant downregulation of P53 gene expression compared to the cisplatin-treated group in Figure 12I. There was no statistically significant difference between the control group and the melatonin group in Figure 12I.

## 4. Discussion

Chemotherapeutic agents are extremely important in combating cancers; however, the prevalence of side effects associated with these drugs is worrisome. Cisplatin is a frontline choice for a variety of malignancies [23]. Only 15–20% of patients respond to its treatment owing to the activation of some survival signaling pathways and inactivation of cisplatin by the thiol-conjugation leading to CP resistance. Hence, increasing the dose becomes necessary to overcome such resistance, affecting normal non-target tissues on the way including, ototoxicity, hepatotoxicity, nephrotoxicity, and reproductive toxicity [24].

Melatonin (N-acetyl-5-methoxytryptamine) is one of the most commonly used supplements among both adults and children [25]. It is the main neurohormone released by the pineal gland and a sleep-wake cycle regulator. It is also a multifunctional hormone that affects most organ metabolism and plays a positive role in healthiness and aging [26].

Cisplatin in the current study significantly retarded the body weight gain and decreased ovarian weight compared to that of normal control rats. These results are in concordance with previous results [27,28]. Cisplatin causes gastrointestinal toxicity and diarrhea and it is a highly emetogenic agent leading to a decrease in appetite [29,30]. In addition, the reduction of the body weight in cisplatin-treated rats might be in part attributed to the direct toxic effect of cisplatin on renal tubules that caused a reduction in water reabsorption and excessive sodium excretion with subsequent polyuria, dehydration, and reduction of body weight [31]. Melatonin co-treatment significantly ameliorates cisplatin-caused body and ovarian weight retardation [32].

Steroidogenesis is an active process in the somatic cells of the ovary to synthesize and secrete sex steroid hormones [33]. Cisplatin increased serum levels of FSH and LH, while reducing both serum levels of E2 and AMH as well as downregulated the ovarian steroidogenic genes levels compared to the control group [34,35,36,37]. In addition, extensive follicular damage resulted in the loss of ovarian steroid hormones with a simultaneous increase of serum FSH and LH [38]. Serum AMH concentration, delicate biomarkers of ovarian damage and reserve, decreased with a low ovarian reserve and follicular destruction [27]. Melatonin co-administration alleviated cisplatin-induced reduction in serum E2 and AMH levels, decreased ovarian steroidogenic genes expression, and increased serum FSH and LH levels [9,35,39,40].

In the current study, a microscopic examination of the ovaries of both control and melatonin groups showed normal histological architecture with the presence of all types of follicles. Histopathological examination of the ovaries of cisplatin-treated group showed a marked loss of normal ovarian architecture with a reduction in ovarian size together with morphometrically reduction in numbers of primordial, growing, and Graafian follicles, and decreased luteal structure size. Severe follicular degeneration was observed in different stages of development with dilated congested blood vessels, hemorrhage, inflammatory cellular infiltrations, and edema obvious in the medulla [41,42]. Melatonin co-treatment attenuated cisplatin-induced microscopic damage where the histological architecture of the ovaries was almost preserved in comparison to the cisplatin-treated group [43,44].

Fibrosis occurs when there is an imbalance in extracellular matrix (ECM) deposition and degradation. Excessive deposition of connective tissues is described as a pathological state and occurs as an outcome of organ injury or failure. TGF-β1 signaling pathway plays an important role in the development of tissue fibrosis by induction expression of several pro-fibrotic genes, such as collagen I, collagen IV, integrin, and fibronectin [45]. In the present study, cisplatin-treated ovaries exhibited an increase in TGF-β1 mRNA expression together with increased collagen deposition in Masson’s trichrome-stained ovarian tissue than that in normal controls [46,47,48].

However, melatonin and cisplatin-cotreated rats exhibited a marked decrease in ovarian TGF-β1 expression and collagen deposition in ovarian tissue. These findings agreed with different studies that reported melatonin administration either intraperitoneally, intravenously, orally, or in drinking water was able to reduce TGF-β expression and decrease tissue and organ fibrosis [49]. Melatonin exerts anti-fibrosis effects by inhibition of epithelial cell injury, including apoptotic and necrotic changes, inflammatory cell infiltration, and the expression of fibrosis-inducing factors, in particular, TGF-β. The activation and proliferation of fibrogenic effector cells, as well as EMT, can also be reduced by melatonin. Moreover, melatonin reduced the deposition of ECM, including glycosaminoglycans and collagen, and the development of fibrosis [50].

In the present study, there were faint PAS reactions and decreased PAS optical density in the zona pellucida surrounding the degenerated oocyte in PAS-stained ovarian sections of cisplatin-treated rats. These results indicated a reduction or even depletion of carbohydrates within the oocytes and their surrounding zona pellucida. The level of PAS staining was markedly decreased in the myocardium and renal cells in cisplatin-treated animals [51,52]. Previous studies have demonstrated that oxidative stress and energy metabolism have numerous connections. Under unfavorable conditions, fibroblasts and lymphocytes increased the rate of glycolysis to maintain ATP production and reduce hypoxia-induced ROS production [53].

However, co-treatment of melatonin and cisplatin, rats exhibited a positive PAS and increased PAS optical density in zona pellucida and oocyte. The previous study showed that melatonin, although by a small amount, corrected thioacetamide, it caused depletion of liver glycogen storage according to PAS staining [54]. The distribution of glycogen density was found to be increased in the bisphenol A and melatonin compared to the bisphenol-A only treated rats [55].

Caspase-3 is the most important executor of apoptosis in the caspase family and represents the final common substrate of both endogenous and exogenous apoptosis pathways. Activated caspase-3 can cleave substrate to induce apoptosis, and therefore, represents an important parameter to reflect apoptosis [56]. In the present study, there were positive reactions and a highly significant increased area percent in caspase-3 immunohistochemically stained ovarian section in cisplatin-treated ovaries. This was concomitant with an increase in the ovarian p53 mRNA expression indicating that cisplatin activates apoptotic processes in ovarian tissue [57,58,59].

However, melatonin decreased levels of ovarian caspase-3 immunoreaction and P53 mRNA expression when co-treated with cisplatin. Melatonin administration significantly ameliorated cisplatin-induced acute kidney injury in mice by decreasing apoptosis by inhibiting the caspase-3, -9, and Bax pathways [60]. In addition, melatonin co-treatment significantly decreased p53, caspase-3, and caspase-9 and increased Bcl-2 and Bal2l1 compared with those in the epirubicin alone [32].

The pathogenesis of CP-induced toxicity has been strongly linked to the induction of oxidative stress damage, the alteration of the tissue antioxidant defense system, and the generation of reactive oxygen species (ROS). Yet, the precise individual mechanism by which CP exerts its harmful effects on different organs of the body is not entirely the same [61]. In the present study, cisplatin led to a highly significant decrease in ovarian levels of GSH, SOD, CAT, and TAC and a highly significant elevation of the ovarian level of MDA compared to the control group [36,62,63]. CP was reported to increase oxidative stress in tissues due to its high reactivity with GSH content, causing its depletion.

Melatonin has significant antioxidant and scavenging effects on free radicals in both the cell cytoplasm and nucleus [64]. Melatonin administration reduced oxidative stress and caused a significant reduction of ovarian MDA levels and a significant elevation of ovarian SOD, CAT, GSH, and TAC when co-administrated with CP [9,40,65]. Melatonin exerted anti-oxidative properties through vigorous free radical scavengers, stimulation of anti-oxidant enzymes, protecting anti-oxidant enzymes against oxidative damage, modulating of several genomic expressions as well as maintenance of mitochondrial homeostasis, as the main source of reactive oxidative species production [7,66].

Inflammation is a defensive reaction of multiple physiological and pathological processes to exert protective mechanisms against external stimuli and tissue damage. NF-κB is a key regulator of apoptosis, immunity, aging, and inflammation owing to its role in the expression of pro-inflammatory genes, including cytokines, chemokines, and adhesion molecules [67,68,69]. Increased levels of TNF-α are considered to be one of the most important markers of increased inflammation [70]. In the present study, CP significantly increased pro-inflammatory (NF-κB, TNF-α, IL-1β, and IL-6) and significantly decreased the anti-inflammatory (IL-10) ovarian mRNA expression [37,71,72].

Meanwhile, melatonin co-treatment reduced the values of pro-inflammatory and significantly increase the anti-inflammatory values in comparison to the CP-treated group. Melatonin significantly suppressed cisplatin elevation of p-NF-kB, TNF-α, and IL1β in the experimental mice’s brains [67]. Melatonin is an immunological modulator that shows pro-inflammatory and anti-inflammatory properties [73]. In early inflammation, melatonin is a temporary pro-inflammatory agent that lasts only 2–3 h and is required for healing to occur in response to acute stress [74]. In late inflammation, melatonin has significant anti-inflammatory properties by inhibiting the binding of nuclear factor κB (NF κB) to DNA, thus decreasing the synthesis of pro-inflammatory cytokines, by reducing cyclooxygenase (Cox), in particular Cox-2, and by suppressing the expression of the inducible gene of nitric oxide synthase [75].

Mitogen-activated protein kinases (MAPKs; Erk1/2, JNK, and p38-MAPK) are involved in several cellular processes including cell progression, differentiation, and apoptosis. They are activated by various extracellular and intracellular stimuli including peptide growth factors, cytokines as (e.g., TNF-α and IL-1β), and extracellular stress (e.g., genotoxicity and oxidative stress) [76,77,78]. MAPK signaling pathways positively regulate the transcription of several inflammatory genes, such as those coding for TNF-α, IL-1β, and COX-2 enzyme together with activation of the transcription factor NF-kB [79]. TNF-α upon binding to its receptors induces the activation of both MAPKs and NF-kB signal pathways leading subsequently to an amplification loop of inflammatory signals inducing further tissue damage [80].

Cisplatin caused a highly significant upregulation of ovarian p38-MAPK and JNK mRNA expression compared to the control group in the current study. Cisplatin has been reported to induce toxicity through the activation of P38 MAPK and JNK pathway [24,81]. Furthermore, co-treatment with melatonin resulted in decreased ovarian p38-MAPK and JNK mRNA expressions significantly compared to CP group. Moreover, melatonin inhibited inflammation and apoptosis in rats with diabetic retinopathy by repressing the MAPK pathway [82]. Exogenous melatonin through MAPK signaling inhibition alleviated inflammatory response in the respiratory tract of asthmatic mice [83].

AMPk, an evolutionarily conserved enzyme, functions as the fundamental regulator of energy homeostasis. Its activation is related to several protective mechanisms including autophagocytosis of damaged cellular structures, alleviation of oxidative stress, inflammation, and physiological suppressor of endoplasmic reticulum stress [84]. AMPK can significantly suppress the levels of pro-inflammatory cytokines; promote the levels of anti-inflammatory cytokines; lead to inhibition of the NF-κB pathways; inhibit MAPK signaling; and decrease cellular ROS [85].

In the current work, cisplatin caused downregulation of ovarian AMPK mRNA expression compared to the control group. Cisplatin has been reported to cause renal injury through the downregulation of AMPK expression [86,87]. In contrast, melatonin co-administration with cisplatin increased the expression of AMPK mRNA in the ovarian tissues. Melatonin treatment upregulated both the AMPK and phosphorylated AMPK in the ovary and different organs [88]. Luan et al. reported that melatonin could relieve tetrabromodiphenyl-ether induced oxidative stress, mitochondrial dysfunction, and apoptosis through the AMPK-Sirt1-PGC-1α axis [89].

PI3K/AKT/mTOR signaling is the central anti-apoptotic intracellular signal transduction pathway regulating cell growth, survival, proliferation, differentiation, and migration. It is involved in the process of oocyte growth, primordial follicle development, and granulosa cell proliferation [90]. Pathway activation leads to phosphorylation and activation of Akt that phosphorylate FOXO3, causing its nuclear export and sequestration together with inhibition of its target gene transcription [91,92]. Upon its inactivation, dephosphorylated FOXO3 translocates to the nucleus where it activates its transcription, transcription of pro-apoptotic factors, loss of mitochondrial membrane potential, cytochrome c release, and caspase activation, and downregulates the levels of Bcl-2 [93,94].

In this work, cisplatin downregulated both ovarian PI3k/AKT/mTOR and PTEN with a highly significant up regulation of FOXO3 mRNA expression compared to the control group. However, in other studies, cisplatin treatment increased PTEN phosphorylation and decreased phosphorylated FOXO3a expression [4,95,96]. This could explain the absence of primordial follicle activation and primordial follicle loss could be related to DNA double-stranded breaks followed by apoptosis in this study. Meanwhile, cisplatin treatment decreased PTEN levels and increased the phosphorylation and activation of key molecules in the PTEN/Akt/FOXO3 pathway causing follicular activation and resulting in premature ovarian failure [97].

Rats treated with CP and melatonin in the present work showed an upregulation of both ovarian PI3k/AKT/mTOR and PTEN with a highly significant downregulation of FOXO3 mRNA expression compared to the CP group. Melatonin has been associated with increased expression of members of the PI3K/Akt/mTOR pathway and suppressed autophagy and apoptosis in polycystic ovary syndrome [98]. Furthermore, melatonin and ghrelin inhibited the cisplatin-induced phosphorylation of PTEN and FOXO3a which induced cytoplasmic translocation of FOXO3a in primordial oocytes [99]. Pretreatment with 30 mg/kg melatonin for 2 h followed by treatment with 2 mg/kg cisplatin for 15 days did not exhibit any protective effect on follicles in the ovarian reserve and granulosa cells of growing follicles in mice [100].

## 5. Conclusions

Our study concludes that melatonin’s antioxidant, anti-inflammatory, and anti-apoptotic activities could modulate ovarian disturbances induced by cisplatin. The findings of our study raise the prospect of melatonin as therapeutic intervention for delaying ovarian dysfunction. Such findings could be an important entry point for preserving fertility while using antineoplastic drugs such as cisplatin used against a wide spectrum of malignancies. Although the therapeutic applications of melatonin should be promising, the side effects of the long-term administration of melatonin remain unclear. Meanwhile, any future clinical application of melatonin should be preceded by rigorous clinical studies.

## Figures and Tables

**Table 1:** Forward and reverse primers sequence of targeted genes.

Gene & Accession Numbers	Forward Primer Sequence (5′ to 3′)	Reverse Primer Sequence (5′ to 3′)
PI3K (NM_053481.2)	CGAGAGTACGCTGTAGGCTG	AGAAACTGGCCAATCCTCCG
Pten (NM_031606.2)	ATACCAGGACCAGAGGAAACC	TTGTCATTATCCGCACGCTC
AKT (NM_033230.3)	GAAGGAGAAGGCCACAGGTC	TTCTGCAGGACACGGTTCTC
mTOR (NM_019906.2)	GCAATGGGCACGAGTTTGTT	AGTGTGTTCACCAGGCCAAA
FOXO3 (NM_017066.3)	AACAAAGGCAGCCTGCTAGT	TCGACGTTGCTGCTGGTATT
TGFβ1 (NM_021578.2)	AGGGCTACCATGCCAACTTC	CCACGTAGTAGACGATGGC
p38-MAPK (NM_019302.1)	TGGAGGTAACCAGGAGGGTT	AAGGCTGTCTTGTCGTAGGC
JNK (NM_053829.2)	TCCAGTTCTCGTACCCGCTA	AGCATGGCGTGACACAGTAA
AMPK (NM_023991.1)	GGCGTGTGAAGATCGGACA	GGCCTGTCAATTGATGTTCTCC
P53 (NM_030989.3)	GTTCGTGTTTGTGCCTGTCC	TGCTCTCTTTGCACTCCCTG
StAr (NM_031558.3)	CCCAAATGTCAAGGAAATCA	AGGCATCTCCCCAAAGTG
CYP11A1 (NM_017286.3)	AAGTATCCGTGATGTGGG	TCATACAGTGTCGCCTTTTCT
CYP17A1 (NM_012753.2)	TGGCTTTCCTGGTGCACAATC	TGAAAGTTGGTGTTCGGCTGAAG
CYP19A1 (NM_017085.2)	GCTGAGAGACGTGGAGACCTG	CTCTGTCACCAACAACAGTGTGG
HSD17B3 (NM_054007.1)	AGTGTGTGAGGTTCTCCCGGTACCT	TACAACATTGAGTCCATGTCTGGCCAG
GAPDH (NM_017008.4)	GGCACAGTCAAGGCTGAGAATG	TGGTGGTGAAGACGCCAGTA

## Data Availability

Data will be provided upon reasonable request.
